# OHMI: the ontology of host-microbiome interactions

**DOI:** 10.1186/s13326-019-0217-1

**Published:** 2019-12-30

**Authors:** Yongqun He, Haihe Wang, Jie Zheng, Daniel P. Beiting, Anna Maria Masci, Hong Yu, Kaiyong Liu, Jianmin Wu, Jeffrey L. Curtis, Barry Smith, Alexander V. Alekseyenko, Jihad S. Obeid

**Affiliations:** 1 University of Michigan Medical School, Ann Arbor, MI 48109 USA; 2 Daqing Branch of Harbin Medical University, Daqing, 163319 Heilongjiang China; 3 University of Pennsylvania Perelman School of Medicine, Philadelphia, PA 19104 USA; 4 University of Pennsylvania School of Veterinary Medicine, Philadelphia, PA 19104 USA; 5 Department of Biostatistics and Bioinformatics, Duke University School of Medicine, Durham, NC 27710 USA; 6 People’s Hospital of Guizhou Province, Guiyang, 550025 Guizhou China; 7 School of Public Health, Anhui Medical University, No 81 Meishan Road, Hefei, 230032 Anhui China; 8 Center for Cancer Bioinformatics, Peking University Cancer Hospital & Institute, Beijing, 100142 China; 9 Pulmonary & Critical Care Medicine Section, Medical Service, VA Ann Arbor Healthcare System, Ann Arbor, MI 48105 USA; 10 University at Buffalo, Buffalo, NY 14260 USA; 11 Department of Public Health Sciences, Medical University of South Carolina, Charleston, SC 29425 USA

**Keywords:** Microbiome, Host-microbiome interaction, Ontology, Ontology of host-microbiome interactions, OHMI, Metadata, OBO Foundry, Rheumatic disease, Rheumatoid arthritis

## Abstract

**Background:**

Host-microbiome interactions (HMIs) are critical for the modulation of biological processes and are associated with several diseases. Extensive HMI studies have generated large amounts of data. We propose that the logical representation of the knowledge derived from these data and the standardized representation of experimental variables and processes can foster integration of data and reproducibility of experiments and thereby further HMI knowledge discovery.

**Methods:**

Through a multi-institutional collaboration, a community-based Ontology of Host-Microbiome Interactions (OHMI) was developed following the Open Biological/Biomedical Ontologies (OBO) Foundry principles. As an OBO library ontology, OHMI leverages established ontologies to create logically structured representations of (1) microbiomes, microbial taxonomy, host species, host anatomical entities, and HMIs under different conditions and (2) associated study protocols and types of data analysis and experimental results.

**Results:**

Aligned with the Basic Formal Ontology, OHMI comprises over 1000 terms, including terms imported from more than 10 existing ontologies together with some 500 OHMI-specific terms. A specific OHMI design pattern was generated to represent typical host-microbiome interaction studies. As one major OHMI use case, drawing on data from over 50 peer-reviewed publications, we identified over 100 bacteria and fungi from the gut, oral cavity, skin, and airway that are associated with six rheumatic diseases including rheumatoid arthritis. Our ontological study identified new high-level microbiota taxonomical structures. Two microbiome-related competency questions were also designed and addressed. We were also able to use OHMI to represent statistically significant results identified from a large existing microbiome database data analysis.

**Conclusion:**

OHMI represents entities and relations in the domain of HMIs. It supports shared knowledge representation, data and metadata standardization and integration, and can be used in formulation of advanced queries for purposes of data analysis.

## Background

A microbiome is defined as a community of microbes (for example, bacteria) found in a particular habitat (for example, a human host) [1–3]. Microbiomes exist in and on human and other hosts, where they are crucial for active immunologic and physiological system development [1, 4–6]. Research in host-microbiome interaction (HMI) has accelerated significantly in the past decade, as evidenced by the rise in the number of microbiome-related publications indexed in PubMed (from 604 to over 11,500 in the ten years since 2018). This growing body of HMI studies and associated data pose significant challenges. For example, it can be difficult for investigators to achieve reproducible results across laboratories, and even more challenging to integrate data systematically across studies. To facilitate advanced data integration and knowledge discovery, several funding sources now require that data generated from funded research be structured to conform to the FAIR (Findable, Accessible, Interoperable, and Reusable) data principles [7]. To support data FAIRness and experimental reproducibility in HMI research, a strategy is needed to standardize the representation of the entities involved in HMI, including host and microbial organisms, microbial locations, and environments. As in other research areas, so also here: the lack of a comprehensive standardized representation of these entities prevents integration and systems-level analysis of the HMI data produced by different studies, laboratories and institutions.

An ontology is a human- and computer-interpretable representation of the types, properties, and interrelationships that exist in a particular domain [8]. Ontologies allow semantically-based reasoning by computer systems, and enable people and machines to make mutually supportive logical inferences. In biomedical research, ontologies have served for some 20 years as powerful tools for data classification, representation of standards, construction of knowledge bases, and enhanced search and analysis. Several microbiology-related ontologies exist, including the NCBI organismal classification (NCBITaxon) [9], the Uberon multi-species anatomy ontology (UBERON) [10] and the Environment Ontology (ENVO) [11]. These ontologies permit standardized representation of, respectively, host and microbial organisms, anatomic locations of microbes inside hosts, and microbiome environments. The Ontology for Microbial Phenotypes (OMP) standardizes phenotypic information relating to microbes [12]. The Ontology of Prokaryotic Phenotypic and Metabolic Characters (MicrO) covers the attributes of prokaryotes, the processes in which they participate, and the material entities (such as cell components, microbiological culture media and medium ingredients) with which they are associated in these processes [13]. Many of the terms in OMP and MicrO were themselves imported from existing OBO ontologies, including the Phenotypic Quality Ontology (PATO) [14], the Gene Ontology (GO) [15], Chemical Entities of Biological Interest (ChEBI) [16], the Protein Ontology (PR) [17], and the Ontology for Biomedical Investigations (OBI) [18].

The above-mentioned ontologies provide components for the systematic representation of certain aspects of HMIs, but they do not cover, for example, HMIs – the interactions between hosts and microbiomes – themselves. They also do not cover the associations between HMIs and specific diseases (such as rheumatoid arthritis), or HMI investigation metadata. We have created the Ontology of Host-Microbiome Interactions (OHMI), therefore, not merely in order to incorporate terms in these specific areas, which are important foci of current microbiome research, but also to provide a single framework for systematic representation of all entities relevant to HMI.

## Methods

### OHMI ontology development

OHMI follows the Open Biological/Biomedical Ontologies (OBO) Foundry (http://www.obofoundry.org/) principles. For example, OHMI satisfies the openness and collaboration principles [19], in that it is based on an open discussion involving representatives from multiple disciplines engaged in microbiome research in which not only the scope of the ontology was identified but also the development strategy, design patterns, and initial use cases. The OHMI GitHub website (https://github.com/OHMI-ontology/OHMI) documents the successive versions of the ontology presented at the 23rd International Scientific Symposium on Biometrics (BioStat 2017), the Sixth Annual Workshop of the Clinical and Translational Science Ontology Group, and the Microbe 2018 meeting of the American Society of Microbiology (ASM).

OHMI uses the eXtensible Ontology development (XOD) methods [20], meaning that it reuses terms from existing ontologies and aligns all terms within a single semantic framework as defined by the Basic Formal Ontology (BFO) [21]. The Ontofox tool was used for extraction and reuse of terms from existing ontologies [22]. The Ontorat tool was used for generating new terms based on consensus ontology design patterns [23]. OHMI was formatted in the Web Ontology Language (OWL2), and the Protégé OWL Editor (version 5.0) [24] was used for manual editing. The HermiT reasoner (http://hermit-reasoner.com/) tool was employed to detect inconsistencies or conflicts arising during development.

### Host-microbiome interaction minimal information collection and ontological representation

All HMI-related data elements were first compiled in a spreadsheet from the literature, public resources, and use cases, then discussed by the community, and transformed into terms and relational expressions for inclusion in the ontology. Following the OBO Foundry principle of reuse, wherever a term was already defined in one or more existing ontologies (identified using Ontobee [25]) we imported the term into OHMI using what we deemed to be the most biologically accurate definition. Otherwise, we created a new term, which was either (1) included in OHMI or (2) suggested for inclusion in an appropriate higher-level OBO Foundry ontology in order to make it available for importing into OHMI.

### OHMI use case studies and evaluation

Our major use case was the study of the association between microbiome profiles and rheumatic diseases. Rheumatic diseases include conditions causing chronic, often intermittent pain affecting the joints and/or connective tissues such as rheumatoid arthritis (RA), ankylosing spondylitis (AS), and systemic lupus erythematosus (SLE). In this study, we manually curated published rheumatic disease-related HMI data from peer-reviewed publications.

We have also defined and used two competency questions derived from the rheumatic disease use case to evaluate the OHMI ontology. For this purpose, we used the Simple Protocol And Resource Description Framework (RDF) Query Language (SPARQL) and Description Logic (DL) languages. SPARQL is a query language that retrieves data stored in the RDF format [26]. SPARQL queries were performed using the Ontobee’s SPARQL endpoint (http://www.ontobee.org/sparql) [25]. The SPARQL scripts are provided in the OHMI GitHub (https://raw.githubusercontent.com/OHMI-ontology/OHMI/master/docs/SPARQL%20scripts.txt). DL queries were performed using the Protégé 5.0 (beta 15) DL Query plugin as described in the Results section.

### Ontology access and license

OHMI is an open source project maintained through https://github.com/ohmi-ontology. The source code, including development and release versions, is available at https://github.com/ohmi-ontology/OHMI. OHMI is released under a Creative Commons 4.0 License. It has been accepted as an OBO library ontology (http://obofoundry.org/ontology/ohmi.html) and deposited in the Ontobee ontology server [25] at http://www.ontobee.org/ontology/OHMI, and in BioPortal [27] at https://bioportal.bioontology.org/ontologies/OHMI. Ontobee is the default server for dereferencing OHMI terms.

## Results

### OHMI ontology design and upper-level structure

**Fig. 1:**
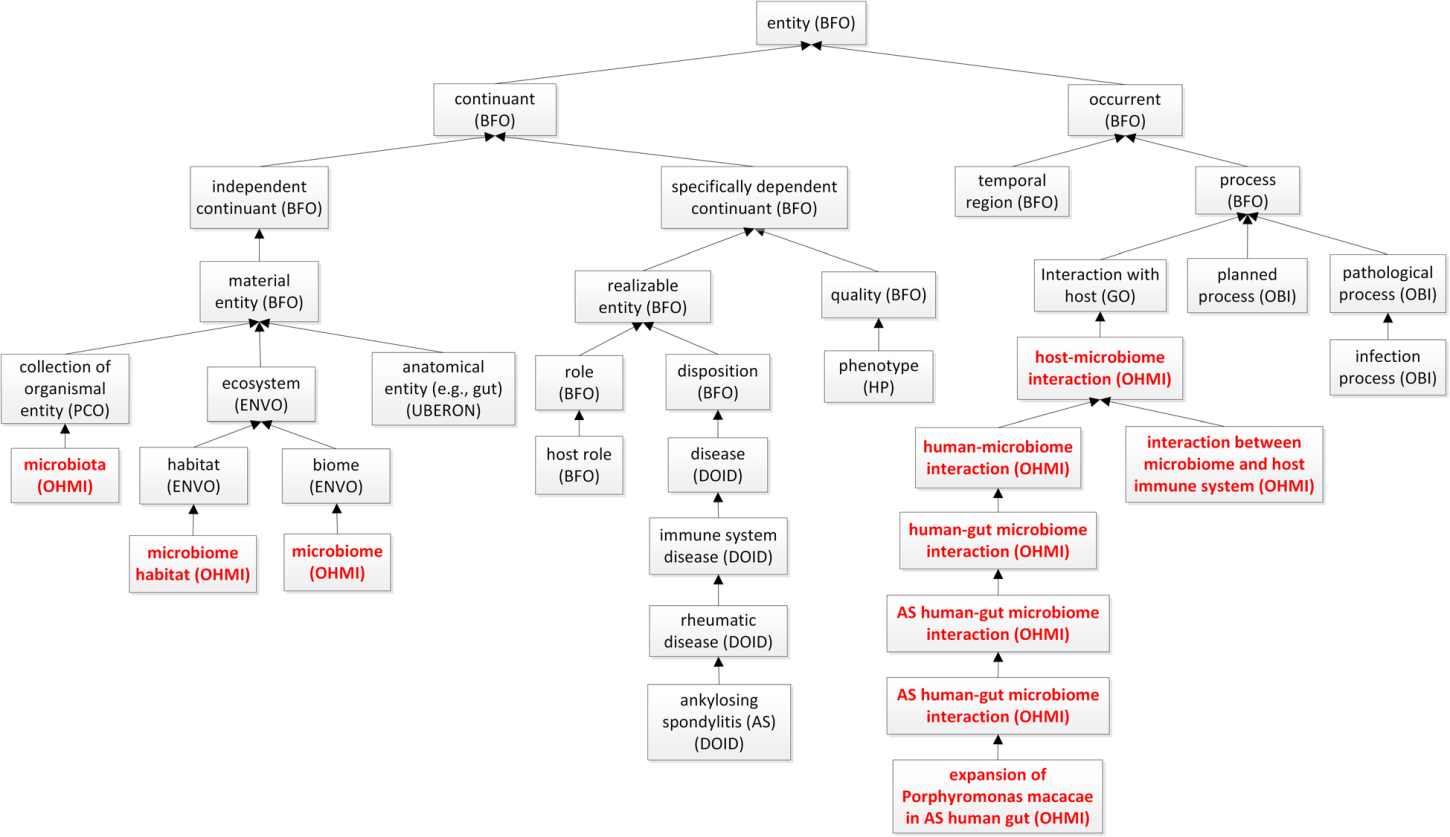
Selected upper level terms and hierarchy of OHMI. OHMI terms are marked by red labels. The full names of listed ontologies are provided in the list of abbreviations at the end of this paper

Figure 1 shows selected upper-level terms and branches of the OHMI hierarchy. Instead of coding everything from scratch, we imported and aligned related terms from existing reference ontologies in the OBO Library, including BFO, NCBITaxon, ENVO, UBERON, OBI, and the Information Artifact Ontology (IAO) [10].

The class OHMI: microbiome is defined as a subclass of ENVO:biome. The latter is defined as follows:

biome = def. an ecosystem to which resident ecological communities have evolved adaptations.

OHMI then defines microbiome as follows:

microbiome = def. a biome that consists of a collection of microorganisms (i.e., microbiota) and the surrounding environment where the microorganisms reside and have evolved adaptations.

OHMI further defines the term ‘microbiota’ as a subclass of the term ‘collection of organisms’ in the Population and Community Ontology (PCO):

microbiota = def. a collection of microbial organisms that reside in a particular environment.

To define the ‘host’ class in OHMI, we first of all define the host role, which is a BFO:role borne by an entity when one or more further entities are spatially located in its interior. An OHMI:host is then an organism that bears a host role in relation to some microbiome.

**Fig. 2:**
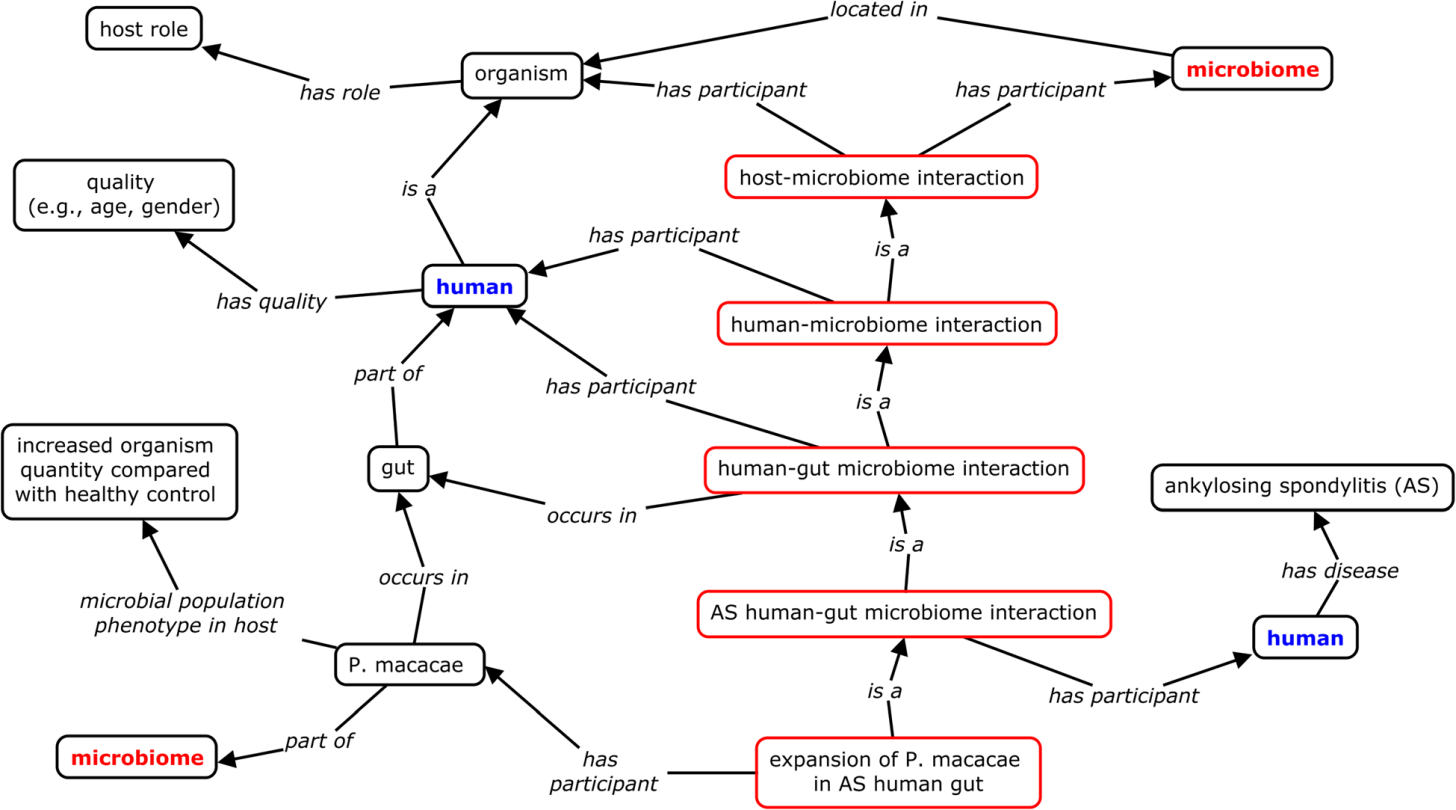
Illustration of OHMI ontology design pattern for representing host-microbiome interactions. The red box represents different levels of host-microbiome interactions. A specific example is the OHMI representation of a human-microbiome interaction in which the human host has the disease ankylosing spondylitis (AS). The human and microbiome classes are duplicated in this figure for clarity. Note that not every organism has the ‘host role’, and the role is here assigned to a host organism only in the case of host-microbiome interactions

The basic design pattern of OHMI is illustrated in Fig. 2. An ontology design pattern is a general pattern to solve a recurrent modeling problem in ontology development by providing scalable and robust representations of entities and entity relations of a certain sort [23]. Terms from the Relation Ontology (RO) [28] have been used to represent OHMI assertions and to formulate corresponding definitions. Specifically, a host-microbiome interaction (HMI) is defined as follows:

host-microbiome interaction = def. an interaction that occurs between a microbiome and its host.

with a logically equivalent class definition as follows:

host-microbiome interaction: interaction and (‘has participant’ some host) and (‘has participant’ some microbiome).

Each HMI occurs in some specific anatomic entity (for example the gut) located in the host organism. This host organism may in addition have a disease – a phenomenon that is illustrated by the representation of a general HMI pattern in patients with ankylosing spondylitis (AS) (Fig. 2). In this example, ‘AS human-gut microbiota interaction’ is a HMI in which the host is a human with AS, while ‘gut’ is the anatomic entity where the microbiota resides. The ‘expansion of *Porphyromonas macacae* in AS human gut’ is an ‘AS human-gut microbiota interaction’ in which the size of the population of *Porphyromonas macacae* is increased (Fig. 2).

As of September 9, 2019, OHMI contains 1238 terms, including 1020 classes, and 128 object properties. OHMI includes 481 OHMI-specific classes and properties with the “OHMI_” prefix, which are new ontology terms not covered in any other OBO Foundry ontologies. More detailed and updated OHMI statistics can be found at the Ontobee statistics page at: http://www.ontobee.org/ontostat/OHMI.

### Systematic collection and representation of rheumatic disease-related HMI knowledge

As a major use case, we systematically collected and annotated the peer-reviewed results of studies of HMI related to rheumatic diseases. Rheumatic diseases are characterized by inflammation of connective tissues, most commonly the joints, but also the tendons, ligaments, bones, muscles, and even solid organs. Our use case study focused on the most common rheumatic diseases, including AS, enthesitis-related arthritis (ERA), gout, psoriatic arthritis (PsA), RA, and systemic lupus erythematosus (SLE), which affect approximately 1% of the global human population. RA is a common rheumatic disease characterized by persistent synovitis, systemic inflammation, and autoantibodies [29]. Many studies have found close associations between rheumatic diseases and HMI [30–33]. Specifically, the gastrointestinal microbiome and its homeostasis are altered in patients with autoimmune and inflammatory rheumatic diseases such as RA [33, 34]. A significant amount of research on the role of the microbiome in autoimmunity has focused primarily on RA [35].

To better understand the relations among rheumatic diseases and microbiomes, we performed a meta-analysis of such relations from relevant literature. In total, from 52 papers (Additional file 2), we found references to 138 bacteria and fungus from the gut, oral cavity, skin, and airway that are associated with the six rheumatic diseases listed above. As an example, the review article by Rosenbaum and Asquith [36] described how microbiome components such as *Prevotella copri, Porphyromonas gingivalis,* and *Collinsella* are expanded or depleted in anatomical locations such as the gut, mouth, lung, and skin in the patients of rheumatic diseases, and the possible underlying mechanisms. These microbe-rheumatic disease associations were represented in the OHMI using the design pattern described above (Fig. 2). Our meta-analysis identified increased or decreased microbe populations in patients with different types of rheumatic diseases (Additional file 2).

**Fig. 3:**
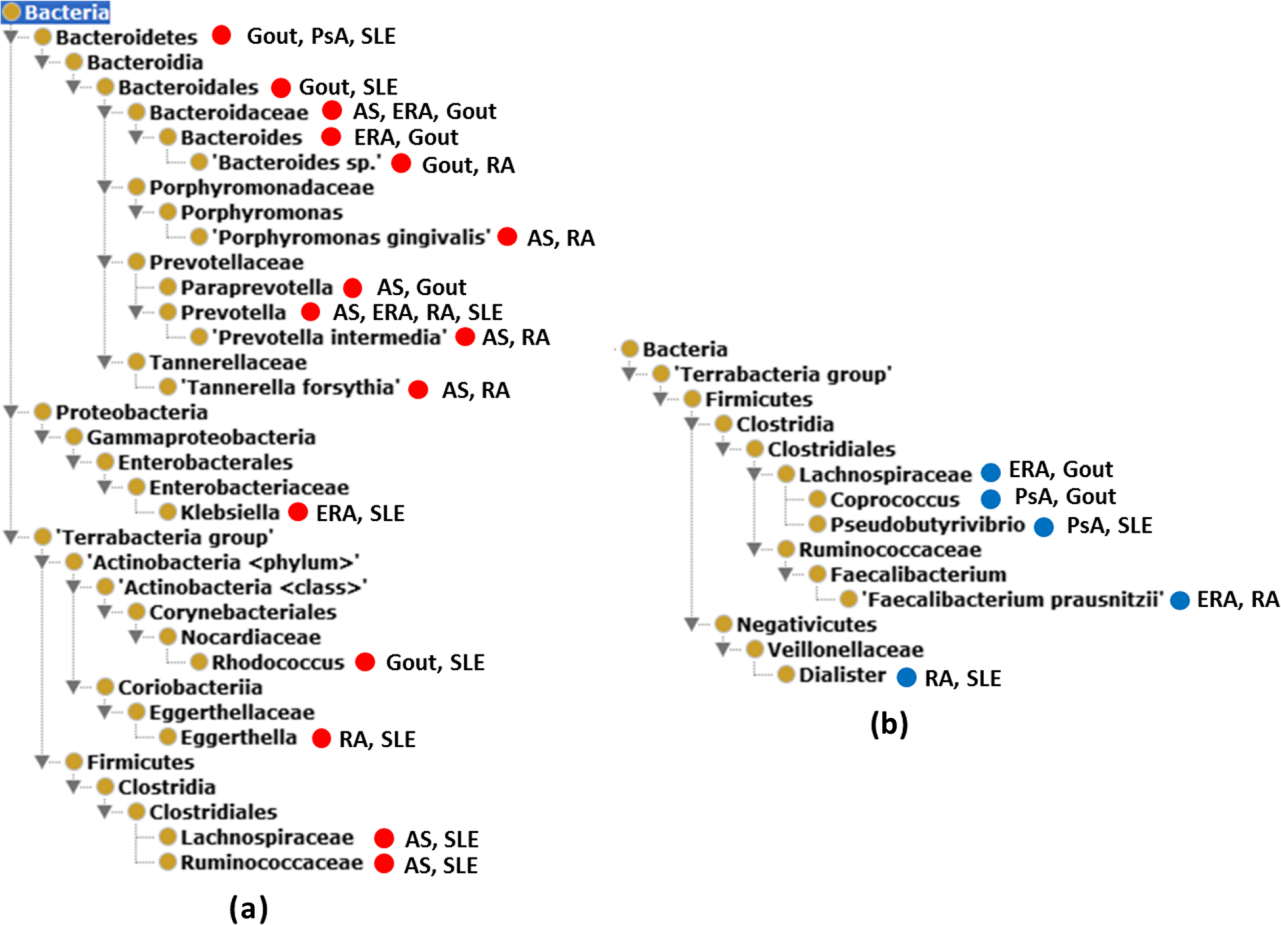
Ontological representation of the bacteria populations increased in the guts of patients with at least two different rheumatic diseases as compared with healthy controls. (**a**) Bacterial population increased in patient guts. (**b**) Bacterial population decreased in patient guts. Many increased and decreased bacterial populations are within the same genus. The red and blue circles represent increased and decreased profiles, respectively. Taxonomy terms without circle and label are used to generate ontological hierarchies

OHMI can also represent specific types of bacteria that are increased in population size in the intestinal microbiota in at least two rheumatic diseases compared to healthy control (Fig. 3). Fifteen different bacterial categories that are enriched in human patients of different rheumatic diseases (Fig. 3a, Additional file 2). Interestingly the phylum *Bacteroidetes* includes 10 out of the 15 bacterial species increased in human rheumatic patients. In comparison, only five bacterial categories are decreased in human patients with rheumatic diseases (Fig. 3b). *Lachnospiraceae* is increased in AS and SLE patients [37, 38] (Fig. 3a), but decreased in ERA and gout patients [39, 40] (Fig. 3b). *Coprococcus* and *Pseudobutyrivibrio*, two bacteria types within *Lachnospiraceae*, were also found to be depleted in rheumatic diseases PsA [41] and gout [39, 41], and SLE [42].

### OHMI representation of key terms in an HMI investigation

A good representative example of the many studies on host-microbiome interactions is the study of the interaction between rheumatic arthritis and human microbiome documented in [43]. In this study, samples were collected from patients before or after antibiotic treatment, and the diversity and composition of the respective microbiome constitutions were identified via 16S rRNA gene sequencing. This study set out to address three key HMI questions: (i) how do healthy and RA patients differ in the compositions of their microbiome? (ii) does the composition of the microbiome in RA patients shift as a result of antibiotic treatment? and (iii) are distinct clinical parameters in RA patients (e.g., autoantibody profiles) associated with distinct microbial community profiles? Analogues of these three questions appear in many microbiome studies across human, animal and environmental disciplines.

**Fig. 4:**
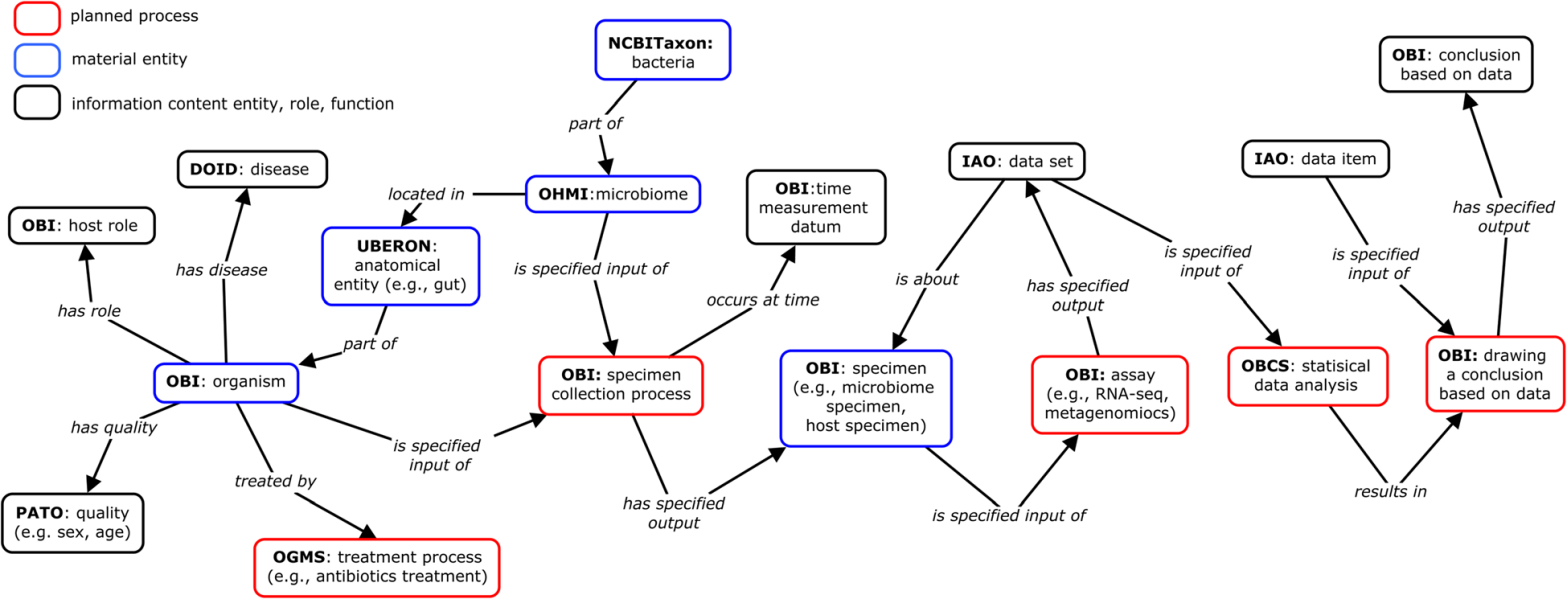
OHMI design pattern of key entities important for HMI investigation. Note that not every organism has the ‘host role’, and the role is here assigned to a host organism only in the case of host-microbiome interaction

To annotate a HMI study as illustrated above [43], we draw on the general OHMI design pattern (Fig. 4). Basically, an HMI investigation involves the collection and analysis of microbiome specimens from an anatomical location in a host organism. The host organism will have a certain demographic profile (sex, age and so forth). It may be either a patient with a specific disease or a healthy control. It may be treated with different interventions, and the samples can be collected at different time points subsequent to the medical intervention. The processed samples are assayed, generating different datasets. Conclusions can then be drawn on the basis of the statistical analysis of these datasets.

The investigation of OHMI host-microbiome interactions involves the representation of various metadata (i.e., “data about data”) types. To standardize these, we reference different resources, starting with the Minimum Information about a Genome Sequence (MIGS) [44] and about any (‘x’) Sequence (MIxS) checklists [45]. While the latter specifies the variables and the information required to describe a genome sequence or any sequence, they do not cover all the information about HMIs and they do not specifically use ontologies. The metadata types documented in these checklist resources were complemented by specific metadata types used in the studies recorded in the peer-reviewed publications such as [43, 46, 47]. These metadata types can be matched to entities defined in ontology.

Table 1 lists a representative minimal checklist of entities that are important for representing an HMI investigation. These terms cover various areas such as host, microbiome sample extraction and analysis, host treatment, and microbiome profile changes. These terms are matched to the metadata types collected for HMI investigation studies. OHMI has imported many corresponding terms from existing ontologies. We also generated many OHMI-specific terms for representation of host-pathogen interactions (Table 1). As a result, OHMI allows us to standardize the representation of a broad range of HMI experiments, and to do this in a way that leads to more advanced integrative data/metadata analyses of such studies, including analyses relating to sequence and other data.

**Table 1:** Selected OHMI entities important for HMI investigation

Topics	Example terms	Ontology
Host	host organism (e.g., human, rat); age, biological sex; disease (e.g., RA, diarrhea); phenotype (e.g., obesity, diarrhea); host anatomical entity (e.g., mouth, stomach); drug product; dysbiosis	NCBITaxon PATO DOID MPO, HPO, … UBERON DRON OHMI
Microbe	microbial taxonomy at various levels (e.g., *E. coli*); species abundance, microbial diversity, microbial genome	NCBITaxon OHMI
Environment conditions	environment (e.g., dwelling, wild field); metabolite (e.g., iron, zinc and arginine), nutrition, …	ENVO CHEBI
Sample collection	collection date/time, collection method, device; geographic location	OBI GAZ
HMI samples	sample from host, e.g., gut, oral, saliva; sample from environment, e.g., soil, table surface	OBI ENVO
Assays	RNA-seq, genome sequencing	OBI
Statistical analyses	ANOVA, t-test, Wilcoxon rank-sum test, MLG-based classifier, KEGG analysis, metagenomic sequencing data, *p*-value	OBCS
HMI results	relative abundance of microbe in host, α-diversity, differentially enriched bacterium (or gene) marker for dysbiosis/disease, overgrowth vs. depletion (or reduced growth); microbiome restoration by treatment (e.g., antibiotics, DMARD)	OHMI (4–5)

The column ‘Ontology’ represents the source ontology in which the example terms are defined. All the terms are defined either in OHMI or imported from other ontologies to OHMI

### Addressing competency questions using OHMI

In addition to the above-mentioned use case of rheumatic disease-associated HMI representation, we also applied OHMI to address several real-life competency questions in additional use cases.

*Competency question 1: What are the human diseases for which a bacterium or bacterial group (for example, E. coli) is expanded in population size in the microbiome*?

A commonly asked competency question relates to the identification of the microbiome components that are increased in humans with a specific disease as compared with healthy control subjects. For example, *Porphyromonas macacae* is expanded in the gut of AS patients, which we represent by means of the term ‘expansion of *Porphyromonas macacae* in AS human gut’ (Fig. 2). This OHMI relationship between the HMI and the bacterium *Porphyromonas macacae* is here represented as:

‘expansion of *Porphyromonas macacae* in AS human gut’: ‘has microbe expanded in diseased host’ some ‘*Porphyromonas macacae’.*

The relational expression ‘has microbe expanded in diseased host’ is an object property that represents the relation between a host-microbiome interaction (HMI) and a microbe, where the population of the microbe is expanded in a diseased host as compared to a healthy host control. As illustrated in the above example, the domain of the relation is a HMI process, and the range is a microbe such as a bacterium. This specific relation is formulated in natural language by means of several different expressions. The inclusion of such a specific relation in OHMI provides a single target for the corresponding annotations which represents a direct logical linkage between a disease-specific HMI and a microbe. OHMI thereby supports efficient knowledge querying and analysis also where we take natural-language inputs as our starting point.

**Fig. 5:**
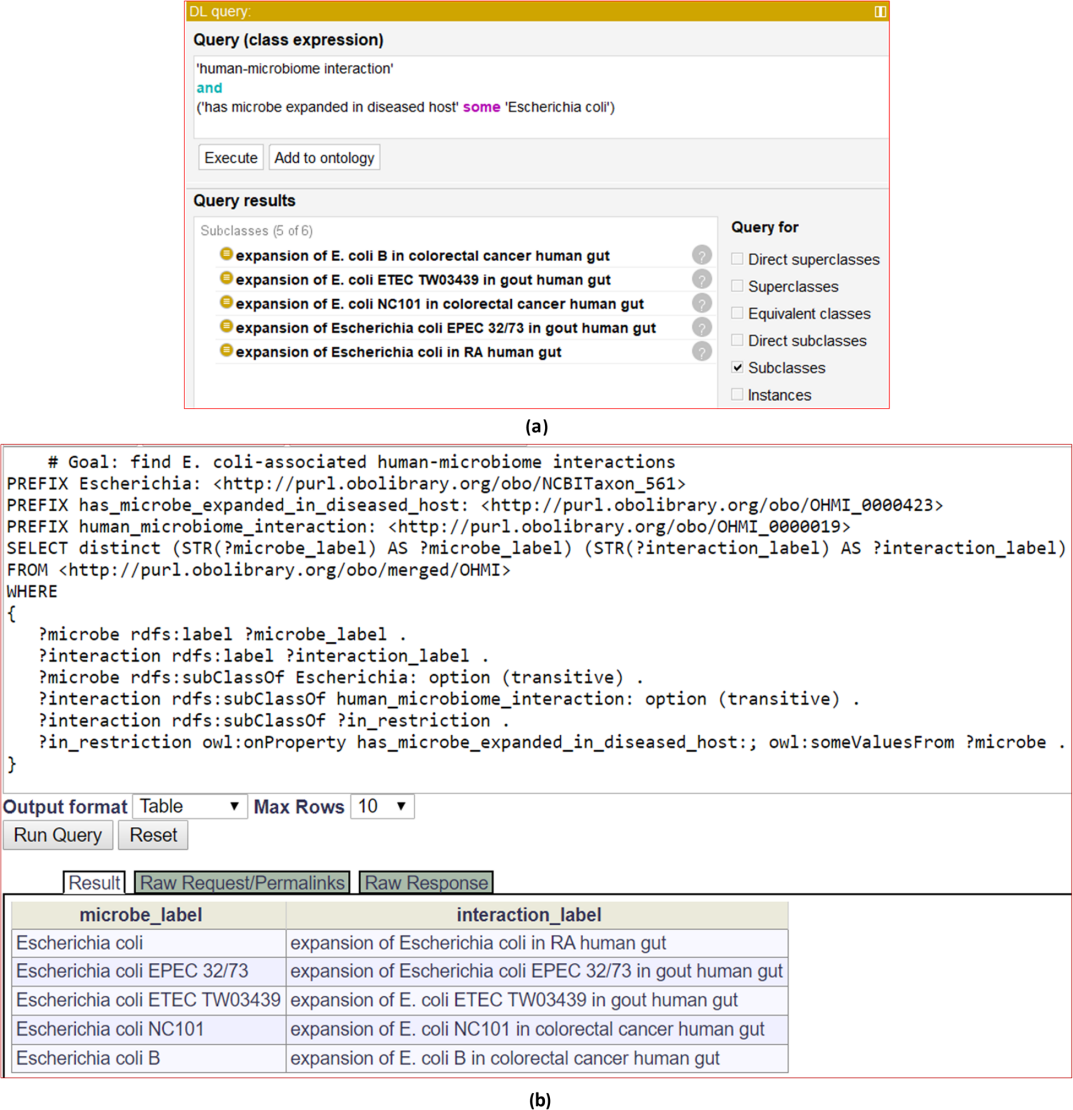
Query of diseases associated with increased *E. coli* in human gut. (**a**) DL query based on the host-pathogen interaction classifications; (**b**) SPARQL query based on the linkage from organism to disease. The SPARQL query was conducted using the Ontobee SPARQL endpoint (http://www.ontobee.org/sparql)

We can use the relationship ‘has microbe expanded in diseased host’ to represent the disease-associated HMIs related to each specific bacterium or bacterium group. For example, such a representation method can be used to identify all the *E. coli*-associated human diseases investigated in [48–51] on the basis of a single DL query, using either Protégé (Fig. 5a) or the Ontobee SPARQL endpoint (Fig. 5). The same outcome was achieved using either approach. The results indicate that *E. coli* and four specific *E. coli* strains were found in rheumatic arthritis, gout, and colorectal cancer.

*Competency question 2: What microorganisms are expanded or depleted in subjects with a specific disease (for example RA) relative to health controls?*

OHMI records manually mined knowledge relating to many types of microorganisms that are increased or decreased in population size in patients with a specific disease relative to healthy controls. This knowledge is often obtained by text-mining all the papers referenced in the literature reports of well-controlled epidemiological studies. OHMI represents this knowledge in two ways. First, it creates a logically well-defined representation of a specific HMI in association with specific disease and microbiome information, as exemplified in Fig. 2 and also shown below:

‘expansion of *Porphyromonas macacae* in AS human gut’: ‘has microbe expanded in diseased host’ some (*‘Porphyromonas macacae*’ and (‘located in’ some (‘lower digestive tract’ and (‘part of’ some (‘*Homo sapiens*’ and (‘has disease’ some ‘ankylosing spondylitis’)))))).

The above is a class definition formulated using logical equivalence. It thus provides necessary and sufficient conditions for the specific HMI class to be exemplified. Using this type of definition, we can specify the link between the microbe *P. macacae* and the disease ankylosing spondylitis (AS). This style of logical definition can serve as a basis for both DL and SPARQL queries. However, since the logical definition includes four relations and is quite complex, it is difficult to write efficient query scripts for queries of this sort.

To solve this problem, OHMI provides shortcut relations to link an organism directly to a disease, for example, ‘has microbe depleted in gut of human with disease’, which is used as follows:

*Prevotella*:

‘microbe susceptibly depleted in gut of human with disease’ some ‘rheumatoid arthritis’.

**Fig. 6:**
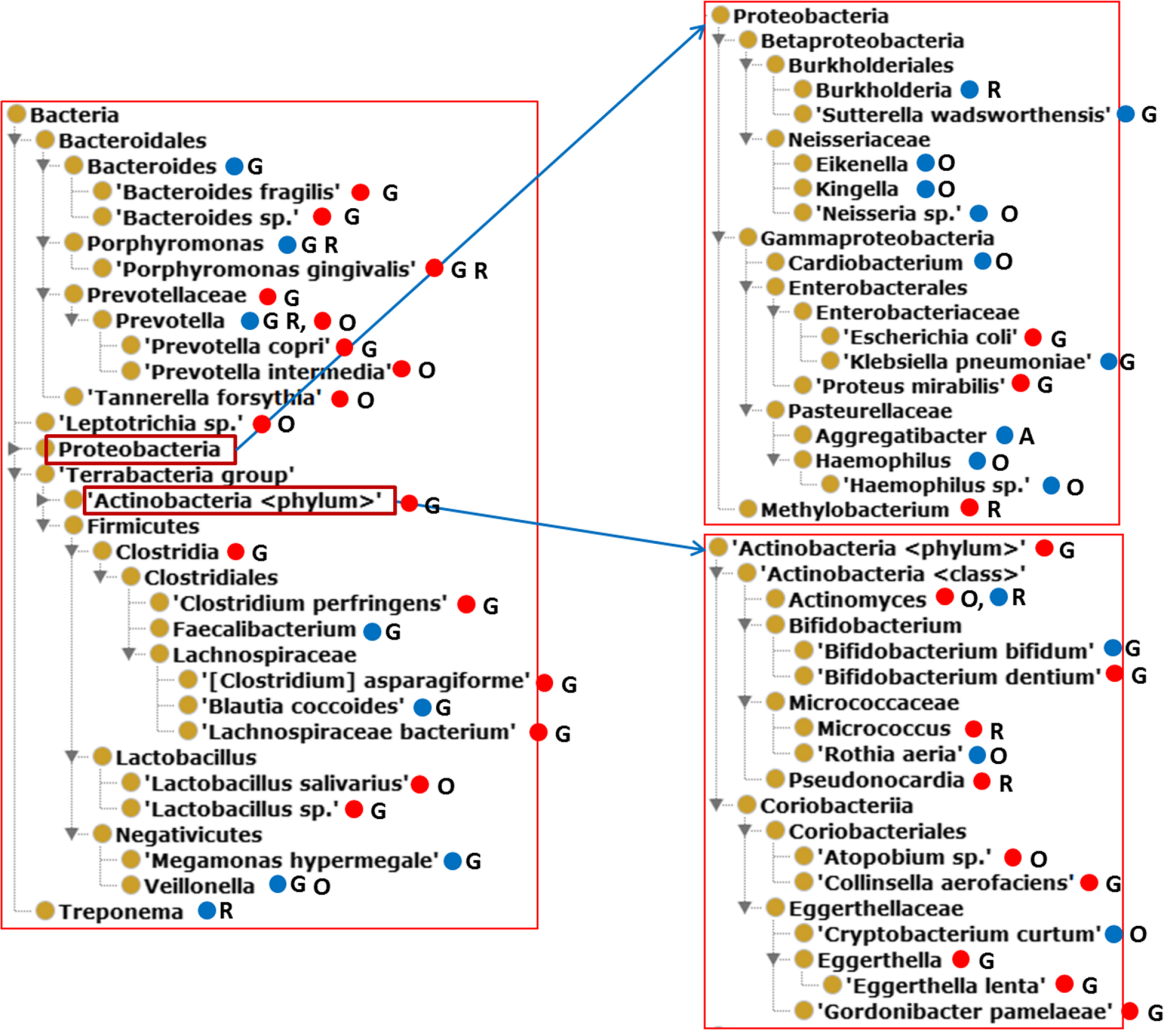
The hierarchy of microbes associated with RA and their profiles. The red and blue circles represent the increased and decreased profiles, respectively. Labeled letters represent locations as follows: G – human gut, O – human oral cavity; R – human respiratory airway. Those taxonomy terms without circle and label are used only to generate the hierarchy

From this we can easily generate DL or SPARQL query scripts. For example, using a SPARQL query using the above relation, we quickly identified 45 distinct bacterial species that are associated with RA through six different relationships (Additional file 1: Figure S1). These 45 species can be organized using an ontological hierarchy (Fig. 6), which reveals several interesting phenomena. For example, both *Lactobacillus* sp. and *Lactobacillus salivariusc* are increased in RA patients, the former in the gut, the latter in the oral cavity. On the other hand, five bacteria under *Betaproteobacteria* were all depleted in RA patients. Among them, three *Neisseriaceae* bacterial groups (*Eikenella*, *Kingella*, and *Neisseria* spp.) are all decreased in the oral cavity in the RA patients, two *Burkholderiales* groups (*Burkholderia* and *Sutterella wadsorthensis*) are depleted in the gut or respiratory airway of the RA patients. Interestingly, *Bifidobacterium bifidum* is decreased in the gut of RA patients; however, *Bifidobacterium dentium* is increased (Fig. 6). These findings merit further investigation to identify the underlying mechanisms associated with each of these phenomena.

## Discussion

This paper introduces the development and application of a community-driven ontology to represent HMIs. OHMI standardizes HMI-related terms and the relations among them. The top level hierarchical structure and general OHMI design pattern are described, followed by descriptions of uses of OHMI with examples and competency questions. OHMI was developed by following best practices [8] and recommended strategies of ontology reuse [7] and design pattern-based development [23]. OHMI seamlessly integrates related terms from existing ontologies with terms specifically related to HMIs. Our use cases demonstrate that OHMI is useful in HMI knowledge representation, data and metadata standardization, and information query and analysis.

OHMI provides a consistent and hierarchical representation of the known HMIs, where the individual microbes, anatomic locations, host species, and host qualities are also represented using the ontology. As shown in the rheumatic disease use case, we were able to identify those expanded and depleted microbes associated with rheumatic diseases. The ontological classification of expanded microbes suggests possible virulent factors and potential pathogenesis in rheumatic diseases, whereas queries identifying depleted bacteria may identify potential candidates for treatment with biological agents [52].

OHMI development is a community effort. For example, OHMI was presented at the Sixth Annual Workshop of the Clinical and Translational Science Ontology Group held in October of 2017 in Ann Arbor, Michigan, USA [53]. The focus of this meeting was to introduce different microbiology-related ontologies, including OHMI, in order to better understand the type of standardization needed to facilitate data integration. There was a consensus that the OHMI and other microbiology-related ontologies should be developed and used by the community to support not only data and knowledge integration but also the design of experimental studies that would be reproducible.

**Fig. 7:**
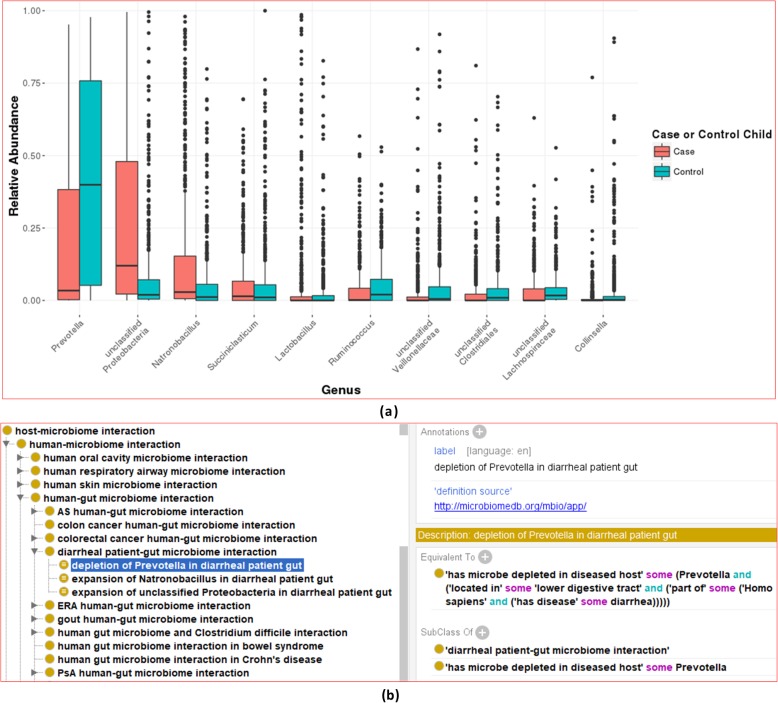
Data mining and ontology representation of microbiome profiles at different species level between diarrhea and health controls. (**a**) MicrobiomeDB data mining. (**b**) OHMI representation of the results

Although the knowledge represented in OHMI is commonly obtained from review of published papers, we can also analyze raw data and generate new information about microbiome and relation examples from an existing database. MicrobiomeDB (http://microbiomedb.org) is a web-based database and systems biology platform for integrating, mining and analyzing data from microbiome experiments [46]. We used the platform to search, analyze and compare microbiome profiles under different conditions. For example, MicrobiomeDB collected over 1000 samples used for study of diarrheal disease and the microbiome in children [54, 55]. Using the dataset collected in MicrobiomeDB and the data analysis tool in the database, we found that *Prevotella* is significantly lower in diarrheal patients compared to controls; however, for unclassified *Proteobacteria* and *Natronobacillus* bacteria the converse was true (Fig. 7a). In addition to the disease condition, age was also found to be a major factor. Our MicrobiomeDB analysis found that the amount of *E. coli* was low in the healthy state in the guts of infants compared to children, but was significantly higher in the infant gut during diarrhea than in the healthy state. The results obtained from analysis of the large MicrobiomeDB dataset can be semantically represented using OHMI (Fig. 7b). This permits the querying of MicrobiomeDB results together with other information drawn from peer-reviewed results. Future directions will include more analysis and representation of MicrobiomeDB data in OHMI in order to improve representation and understanding of HMI mechanisms.

Reproducibility of both experiments and data analyses depends on consistent procedures employed across different settings. OHMI harmonizes the metadata representation of the interactions between microbiomes and hosts along with associated biological processes, supporting the integration and systems-level analysis of HMI data produced by different laboratories and institutions. To support reproducibility and advanced analysis, we plan to work with the MicrobiomeDB project to support ontology-based data standardization, browsing, and advanced analyses. Specifically, MicrobiomeDB will use OHMI terms and metadata types to standardize the currently still highly heterogeneous MicrobiomeDB data. The information captured in OHMI can also provide prior knowledge that can be used to enhance queries and analyses of MicrobiomeDB data. One such strategy is to allow users who have identified differentially abundant taxa (using the existing differential abundance app) to leverage OHMI to ask whether the identified list of differentially abundant taxa is enriched for any disease processes or interactions. Such an approach is similar to how the Gene Ontology (GO) has been used to support data analysis, by providing prior knowledge relating to the roles of given genes in realizing given functions, knowledge which can then be used to support gene enrichment and other data analysis [56]. Such a strategy has its advantages over alternatives such as simply using relational database representations, since the ontology-based approach supports better standardization, flexibility, interoperability, machine interpretation, and extensible tool development.

A newly funded project is to apply OHMI to study the host-microbe interactions related to gastric cancer. Gastric cancer (GC) is the fifth most prevalent malignancy and the third leading cause of cancer death worldwide. Almost half of new cases occur in China, and it is the second leading cause of cancer death in China. The strongest risk factor for gastric cancer is chronic *Helicobacter pylori* infection. People with an *H. pylori* infection have a roughly six-fold greater risk of developing gastric cancer than uninfected people. However, not all people infected with *H. pylori* have gastric cancer, suggesting that there are more factors and mechanisms involved in gastric carcinogenesis which are not yet understood. Comparative bacterial genomic analysis in patients with or without gastric cancer allows gene-level study of host-microbiome interactions as it relates to gastric carcinogenesis in humans. Clinical trials together with multi-omics studies are being performed to further explore the mechanisms of host-microbiome interactions leading to gastric cancer.

OHMI is an ongoing project. Future work includes expansion of OHMI to cover more diseases such as obesity and inflammatory bowel disease. More molecular and cellular entities and processes will be included to better understand HMI mechanisms. Such host-specific microbiome profiles may serve as a biological marker for specific host types or specific health conditions. Various types of biological conditions (for example associated with patient age, biological sex, genetic makeup, and environment) may affect the outcomes of HMIs and will be systematically studied. Thus, one important outcome of OHMI is to identify opportunities to collect microbiome data related to pervasive exposures that cut across multiple disease states (for example tobacco smoking and Western versus traditional diets) and which were not captured by the Human Microbiome project. OHMI is expected to become an ontology-based interoperable knowledge base of host-microbiome interactions, which can be used to address many technical challenges in constructing microbiome-disease association knowledge bases [57] and thereby help to solve fundamental scientific questions. We also welcome researchers interested in the topic to participate in the community-based OHMI development and its application to other use cases.

## Conclusion

OHMI ontologically represents entities of various types associated with HMIs and the relations among such entities. Our use cases demonstrate how OHMI could be used as a canonical platform for HMI knowledge representation, metadata standardization, semantic querying and data analysis.

## Supplementary information

**Additional file 1:** **Figure S1.** SPARQL query for all microbes associated with RA and their relations. The query was conducted using the Ontobee SPARQL endpoint

**Additional file 2:** Manually annotated rheumatic disease HMI data from the literature. The annotated information is represented in OHMI

## Data Availability

All data generated or analyzed during this study are included in this published article and Additional files.

## Abbreviations

AS: Ankylosing spondylitis
BFO: Basic Formal Ontology
ChEBI: Chemical Entities of Biological Interest
DL query: Description Logics query
DMARD: Disease-modifying anti-rheumatic drug
DOID: Disease Ontology
DRON: Drug Ontology
ERA: Enthesitis-related arthritis
GO: Gene Ontology
IAO: Information Artifact Ontology
MicO: Ontology of Prokaryotic Phenotypic and Metabolic Characters
MPO: Mammalian Phenotype Ontology
NCBITaxon: NCBI organismal classification
OBCS: Ontology of Biological and Clinical Statistics
OBI: Ontology for Biomedical Investigations
OBO: The Open Biological and Biomedical Ontologies
OHMI: Ontology of Host-Microbiome Interactions
OMP: Ontology for Microbial Phenotypes
OWL: Web Ontology Language
PATO: Phenotypic Quality Ontology
PR: Protein Ontology
PsA: Psoriatic arthritis
RA: Rheumatoid arthritis
RDF: Resource Description Framework
SLE: Systemic lupus erythematosus
SPARQL: SPARQL Protocol and RDF Query Language
UBERON: Uberon multi-species anatomy ontology
