# Supplementary material for: OHMI: the ontology of host-microbiome interactions
Source: J Biomed Semantics. 2019 Dec 30;10:25. doi: 10.1186/s13326-019-0217-1 (PMC6937947; doi:10.1186/s13326-019-0217-1)
Supplement: Supplementary file 1 — Additional file 1: Figure S1. SPARQL query for all microbes associated with RA and their relations. The query was conducted using the Ontobee SPARQL endpoint [file 13326_2019_217_MOESM1_ESM.docx]

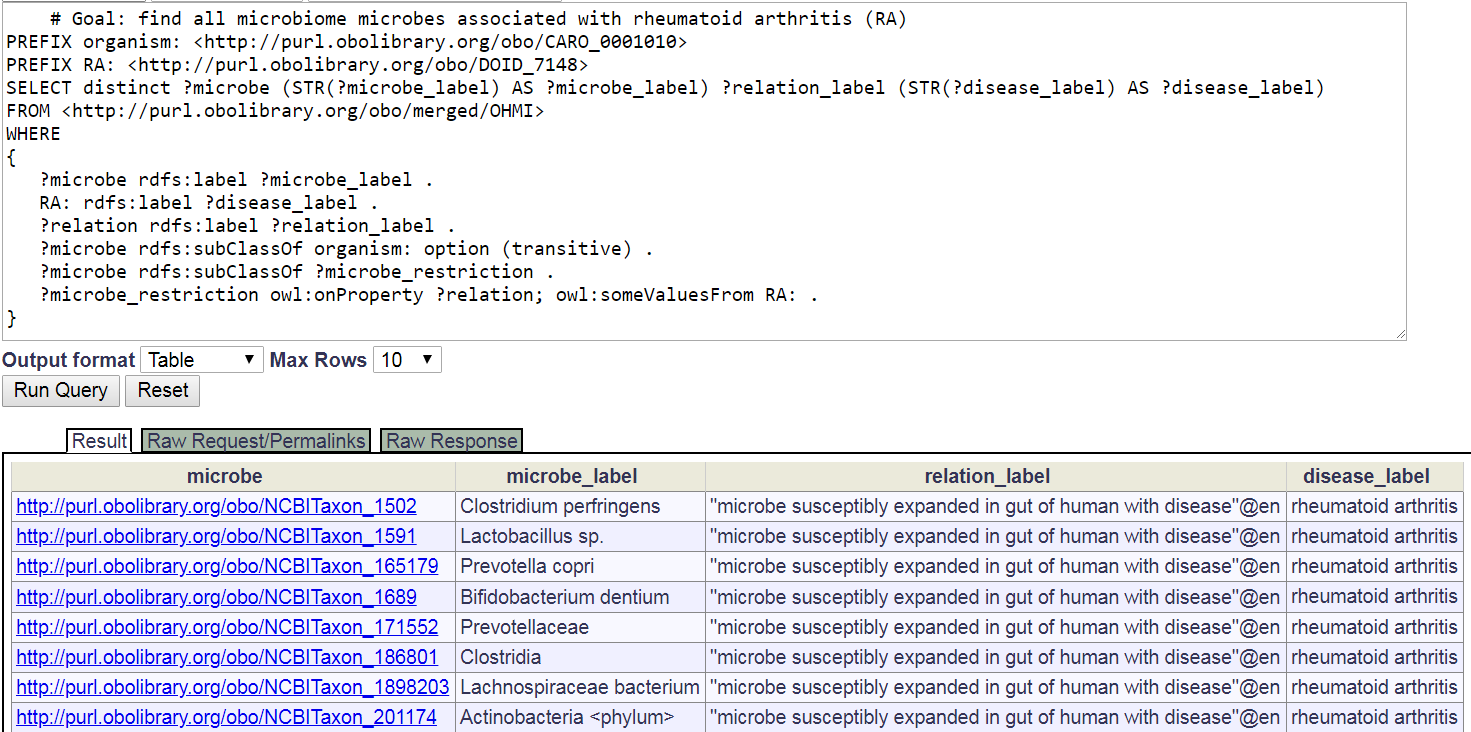


**Supplemental Figure 1.** **SPARQL query for all microbes associated with RA and their relations.** The query was conducted using the Ontobee SPARQL platform.
